# Adherence, quality of life and number of daily pills in a large cross-sectional study

**DOI:** 10.1186/1758-2652-13-S4-P116

**Published:** 2010-11-08

**Authors:** N Gianotti, L Galli, B Bocchiola, T Cahua, P Panzini, D Zandonà, S Salpietro, M Maillard, A Danise, A Pazzi, A Lazzarin, A Castagna

**Affiliations:** 1 San Raffaele Scientific Institute, Infectious Diseases, Milano, Italy

## Purpose of the study

To assess if with current cART regimens the number of daily pills is still a determinant of adherence and quality of life (QOL).

## Methods

Cross-sectional study of patients (pts) on cART followed at our centre. An adherence and QOL questionnaire was offered between March and May 2010 to all pts at drug supply; both parameters were evaluated by visual scale. Results are described as median (IQR) or frequency (%). Linear correlation was evaluated by the Spearman correlation coefficient. Generalized linear model (GLM) was applied considering adherence or QOL as alternative outcome variables.

## Results

2114 pts [aged 46.3 (41.9-51.1) years, 448 (21.3%) females, infected since 13.7 (8.0-19.2) years, treated with antiretrovirals since 11.5 (5.4-14.1) years, 260 (12.4%) of whom with a previous diagnosis of AIDS] were included in the analysis.

At the time of survey, 1793 (87%) had <50 HIV RNA copies/mL and CD4+ were 570 (403-746)/µL. Adherence and QOL were 100 (100-100)% and 83 (61-100)%, respectively; the number of daily pills in the ongoing regimen was 3 (3-5); 914 (43.2%) pts were receiving a BID and 1200 (56.8%) a QD regimen. At univariate analysis (figure), adherence was correlated to QOL but not to the number of daily pills, whereas QOL was weakly and inversely related to the number of daily pills; both adherence and QOL were not different between pts receiving a BID or QD regimen.

At GLM, after adjustment for age, gender, HIV risk factor, current CD4+, number of pills or dosing interval, adherence was associated with current HIV RNA [adjusted mean±SE: 94.3±0.48% for pts with <50 copies/mL vs 88.6±1.10% for those with ≥50 copies/mL, p<0.0001], gender [90.4±0.94% for females vs 92.5±0.60% for males, p=0.023] and current CD4+ (β=0.003 p<0.001).

When adjusting for the same variables, QOL was higher in pts with undetectable viremia (78.7±0.7% vs 74.0±1.58%; p=0.004), in males (78.3±0.89% vs 74.5±1.39%; p=0.009), in MSMs vs heterosexuals vs others [78.5±1.37% (p<0.0001) vs 77.2±1.33% (p=0.008) vs 73.3±1.07% (reference group)], and also associated with age (β=-0.25, p<0.0001), current CD4+ (β=0.011, p<0.0001), and the number of pills (β=-0.77, p=0.019), but not with dosing interval.

**Figure 1:**
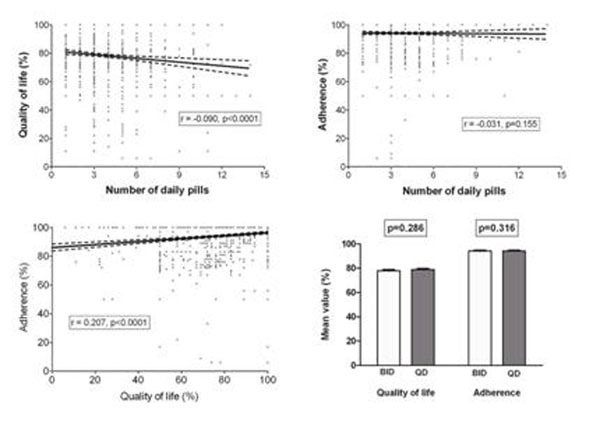


## Conclusions

In this highly adherent population, adherence was not associated with the number of daily pills or dosing interval. QOL was associated with the pill burden, but the pill burden explained <1% of QOL. Both adherence and QOL were strongly associated with virological response.

